# Limited Added Value of Magnetic Resonance Imaging After Dynamic Transvaginal Ultrasound for Preoperative Staging of Endometriosis in Daily Practice: A Prospective Cohort Study

**DOI:** 10.1002/jum.14783

**Published:** 2018-09-23

**Authors:** Judith P. Berger, Johann Rhemrev, Maddy Smeets, Onno Henneman, James English, Frank W. Jansen

**Affiliations:** ^1^ Bronovo Hospital Bronovolaan the Netherlands; ^2^ Leiden University Medical Center Leiden the Netherlands; ^3^ Technical University of Delft Delft the Netherlands

**Keywords:** diagnosis, endometriosis, gynecology, magnetic resonance imaging, transvaginal ultrasound

## Abstract

**Objectives:**

To assess the added value of magnetic resonance imaging (MRI) after dynamic transvaginal ultrasound (TVUS) in the diagnostic pathway for preoperative staging of pelvic endometriosis.

**Methods:**

A prospective observational study was conducted between April 22, 2014, and May 1, 2015. During that period, 363 patients with a clinical suspicion of endometriosis were included. All patients underwent a history, clinical examination, and dynamic TVUS examination. Most of the patients (n = 274) underwent conservative treatment according to the European Society of Human Reproduction and Embryology guidelines. Eighty‐nine patients were selected for surgery, of whom 72 patients underwent the complete diagnostic pathway: ie, history, clinical examination, dynamic TVUS, and MRI. All data were analyzed by the nonparametric McNemar test for comparing each step in the diagnostic algorithm.

**Results:**

The sensitivity and specificity for the history, pelvic examination, and dynamic TVUS were 93.7% and 55.6% (*P* < .001), respectively; when MRI findings were included, the sensitivity and specificity were 85.9% and 62.5%. Adding MRI routinely to the diagnostic procedure of endometriosis did not significantly improve the sensitivity or specificity.

**Conclusions:**

There is no significant added value of routine MRI after dynamic TVUS for the preoperative staging of endometriosis.

**Abbreviations**
AFS: American Fertility Society
MRI: magnetic resonance imaging
NPV: negative predictive value
PPV: positive predictive value
TVUS: transvaginal ultrasound
US: ultrasound

Endometriosis is defined as the presence of endometriotic glands and stroma outside the uterus. Three types of endometriosis have been defined histologically: peritoneal, ovarian, and deep infiltrating endometriosis, the latter being defined as infiltrating greater than 5 mm into the surrounding tissues.^1^ The exact prevalence of endometriosis is not known but is estimated to be 2% to 10% in premenopausal woman and up to 47% in infertile woman.^2^, ^3^

The definitive diagnosis of endometriosis is made by laparoscopy. However, there is often a delay between the onset of symptoms and the final diagnosis: 6.7 to 11.7 years with a mean of 8.5 years has been reported.^4^, ^5^, ^6^

For the evaluation of deep infiltrating endometriosis, various diagnostic procedures have been investigated in the last decade: transvaginal ultrasound (TVUS), magnetic resonance imaging (MRI), and virtual colonoscopy.^7^, ^8^, ^9^, ^10^ Clinical examination and TVUS are widely used as first diagnostic tools, whereas the less‐accessible MRI is used for assessing the severity of the disease.^8^, ^11^

Both TVUS and MRI proved to be highly sensitive in detecting deep infiltrating endometriosis. Transvaginal US has 91% sensitivity and 98% specificity for detecting endometriosis in the bowel.^12^, ^13^ For preoperative staging, MRI is frequently used and has sensitivity of at least 75% and specificity of 80% or higher for different anatomic sites in the pelvis.^11^ Abrao et al^9^ compared clinical examination, TVUS, and pelvic MRI in the preoperative diagnosis of deep infiltrating endometriosis and concluded that TVUS had better sensitivity and specificity over MRI.

Transvaginal US is operator dependent but highly sensitive in experienced hands. It is readily available and can show either fixity or mobility of pelvic organs as well as identifying the location of maximum patient tenderness; however, lesions outside the pelvis are not visible. Magnetic resonance imaging is less operator dependent but is also less sensitive in detecting bowel endometriosis because of movement artifacts. Furthermore, MRI is less accessible than US.^8^

Combining different US features such as the uterine sliding sign, hard and soft markers (eg, hydrosalpinx and loculated fluid), and the mobility of pelvic organs with tenderness‐guided US results in a more dynamic TVUS diagnostic tool and may very well be suited for identifying deep infiltrating endometriosis.^14^, ^15^, ^16^, ^17^ The aim of our study was to evaluate the added value of MRI after dynamic TVUS in the diagnosis of both endometriosis and deep infiltrating endometriosis for preoperative staging of pelvic endometriosis.

## Materials and Methods

A prospective observational study was conducted in our referral center for endometriosis between April 22, 2014, and May 1, 2015. Exclusion criteria comprised patients younger than age 18 years, patients for whom dynamic TVUS was not possible (eg, Virgo condition), and patients with claustrophobia or contraindications to MRI. A total of 363 patients with a clinical suspicion of endometriosis were included. The local Ethical Committee approved the study as exempt from review because this study had no impact on routine patient care.

**Figure 1:**
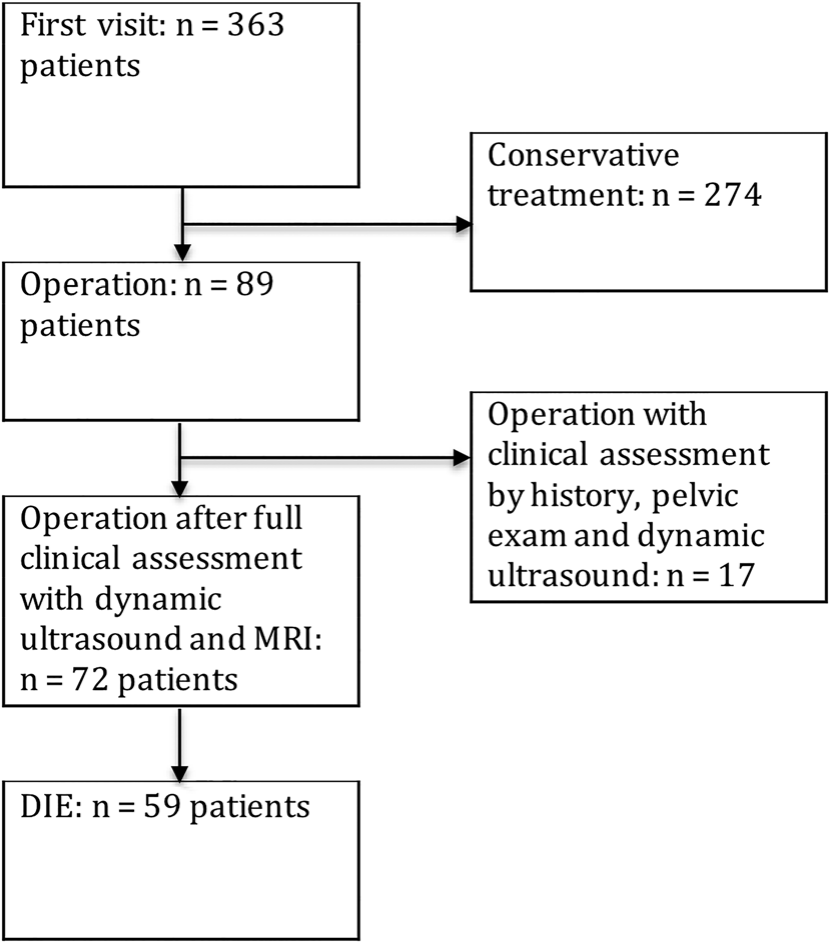
Study flowchart. DIE indicates deep infiltrating endometriosis.

All patients underwent a history, clinical examination, and dynamic TVUS examination. After each step in the diagnostic pathway, the extent and severity of the endometriosis were determined. Most of the patients (n = 274) underwent conservative treatment according to the European Society of Human Reproduction and Embryology guidelines (Table 1).^18^ Finally, 89 patients were selected for surgery, of whom 72 patients underwent the full diagnostic pathway: ie, history, clinical examination, dynamic TVUS, and MRI (Figure 1).

**Table 1:** Conservative Treatment in Patients With Signs of Endometriosis (n = 274)

Treatment	n (%)
Physical therapy	24 (8.7)
Pain consultant	18 (6.5)
Hormonal treatment	111 (40.5)
Dietician	23 (8.4)
Psychologist	36 (13.1)
Combination of treatments	62 (22.5)

### *History and Clinical Examination*

The history included symptoms of dyspareunia, dysmenorrhea, dysuria, dyschezia, and cyclic or chronic pelvic pain and subfertility. In addition, patients were questioned about the quality of their social life: ie, physical discomfort and depression (Table 2).

**Table 2:** History Checklist

Item	Questions
Reason for referral	By whom are you referred?
	Has endometriosis already been diagnosed? By whom?
History	Were you ever operated on?
	Did you ever have any unexplained abdominal pain for which you went to a hospital?
Symptoms	Age at menarche?
	Have your symptoms started at menarche, or did they start later? At what age?
	Where do you have pain (abdomen, lower back, legs, shoulder)?
	When during your menstrual cycle do you have the pain?
	Are you taking a contraceptive pill?
	Do you use any pain medication?
Children	Do you have any children?
	If yes, did you get pregnant very easily? How did you deliver?
	If no, do you desire to get pregnant? Did you already try to get pregnant?
Social/work	Are you frequently absent from work because of your symptoms?
	Do you need to cancel social appointments because of your symptoms?
Psyche	Do you feel fatigue?
	Do you have mood swings?
	Do you feel depressed?
	When in your menstrual cycle do you have these feelings?
Micturition	Do you have any difficulties or pain with micturition?
	Does it feel different during menstruation?
	Do you have a residual feeling?
	Did you see any blood in your urine?
Defecation	Do you have any difficulties or pain with defecation?
	Does it feel different during menstruation?
	Do you have obstipation or diarrhea?
	Do you lose blood with defecating?
Sexual	Do you have pain during or after sexual intercourse? Deep or superficial?
	Do you lose blood after sexual intercourse?

Physical examinations were performed by 2 examiners, both with more than 15 years of experience in endometriosis (M.S. and J.R.). Patients underwent a pelvic examination focusing on blue spots on the cervix, vaginal wall involvement, and the posterior vaginal fornix. Mobility of the uterus and ovaries, uterosacral ligaments, and nodules in the pouch of Douglas or anterior fornix was assessed by palpation. Suspected deep infiltrating endometriosis was noted separately.

### *Dynamic TVUS*

Based on the information obtained during the history and pelvic examination, a dynamic TVUS examination was performed by a single examiner with 5 years of specialization in US for endometriosis (J.P.B.) using a transvaginal transducer at a frequency of 5–9 MHz (Voluson E8; GE Healthcare, Buckinghamshire, England). No bowel preparations or vaginal contrast agents were used; the bladder needed to be partially filled.

First, a standard evaluation of the uterus and ovaries was performed. Then, the dynamic part of the examination was conducted: the uterine sliding sign, tenderness‐guided US, and evaluation of hard and soft markers.

The uterine sliding sign establishes whether the rectum moves freely across the posterior wall of the uterus, cervix, or both when pressed gently with the vaginal transducer. If the sign is negative (ie, the rectum or rectosigmoid does not slide freely across the uterus or cervix), the pouch of Douglas is considered obliterated.^14^, ^15^

Tenderness‐guided US exploits the fact that endometriotic nodules can evoke pain. Therefore, patients can indicate which points are painful during the examination. These sites are then examined with extra attention.^16^, ^17^

Hard markers are structural abnormalities: ie, hydrosalpinx or an endometrioma. Soft markers are loculated peritoneal fluid, ovarian mobility, and site‐specific tenderness.^18^ Hypoechoic nodules were actively looked for in the bowel, bladder, sacral uterine ligaments, posterior vaginal fornix, and paraureteral area as described by Bazot et al^7^ and noted separately.

### *Magnetic Resonance Imaging*

Magnetic resonance imaging was performed within 6 weeks after dynamic TVUS. The MRI examinations were performed on a 1.5‐T superconducting magnet (Magnetom Avantofit; Siemens AG, Erlangen, Germany) using an 18‐channel radiofrequency body coil. The MRI protocol consisted of multiplanar turbo spin echo T2‐weighted images (512 matrix; axial, sagittal, and coronal with a voxel size of 0.8 × 0.8 × 4.0 mm) and axial and sagittal T1‐weighted fat‐saturated breath hold sequences (320 matrix; voxel size of 1.3 × 1.3 × 6.0 mm. Twenty minutes before the MRI, patients were administrated 20 mg of butylscopolamine bromide intravenously (Buscopan; Sanofi‐Aventis, Paris, France) for bowel movement inhibition. No enema was administered; no vaginal distention was applied; and patients did not fast. No contrast agent was used. All MRI examinations were evaluated by a single radiologist with 10 years of experience in endometriosis. The radiologist was blinded to the results of the history, clinical examination, and dynamic TVUS.

The diagnosis of deep infiltrating endometriosis was made essentially as described by Bazot et al^7^ if one of the following criteria was met: hyperintense foci on the fat‐suppressed T1‐weighted images with corresponding hemorrhagic foci on T2‐weighted images, areas of fibrosis in the pelvic region, distortion of normal anatomy without any other explanation, and discontinuation of normal fatty tissue between organs.

### *Surgical and Histologic Findings*

All patients included (n = 72) underwent laparoscopic resection of all endometriosis. Staging of endometriosis was determined by 2 gynecologists on visual inspection at laparoscopy according to the revised American Fertility Society (AFS) criteria; deep infiltrating endometriosis nodules were noted separately.^19^ All visual diagnosis of endometriosis was confirmed by a histologic examination, as visual diagnosis does not correlate well with pathologic findings.^20^

### *Statistical Analysis*

All analyses were performed with SPSS version 23 software (IBM Corporation, Armonk, NY). The sensitivity, specificity, positive predictive value (PPV), and negative predictive value (NPV) were calculated for each step in the diagnostic process. The nonparametric McNemar test was used for comparing between each step in the diagnostic algorithm. *P* < .05 was considered statistically significant.

## Results

The characteristics of the 72 patients are summarized in Table 3. All 72 patients underwent surgery; in 59, deep infiltrating endometriosis was confirmed; and 13 patients had a diagnosis of “low‐grade endometriosis.” With respect to the surgical location, all patients had peritoneal endometriosis; 34 patients had bowel endometriosis; 3 patients had bladder endometriosis; 3 patients had endometriosis around the ureter; and 26 patients had an ovarian endometrioma.

**Table 3:** Clinical Characteristics of the Patients (n = 72)

Characteristic	Value
Age (range), y	36.3 (22–55)
Dysmenorrhea, %	82.8
Dyschezia, %	70.7
Dysuria, %	40.0
Dyspareunia, %	65.5
Previous surgery for endometriosis, %	41.2

### *Comparison of the Consecutive Steps: History, Clinical Examination, Dynamic TVUS, and MRI in the Diagnosis of Endometriosis*

The sensitivity, specificity, PPV. and NPV for each consecutive step in the diagnosis of pelvic endometriosis are given in Table 4. Notably, the sensitivity after adding the results from the history, clinical examination, and dynamic TVUS was 93.7% (*P* < .001). When the MRI results were included, the sensitivity was 85.9% (*P* = .219). However, after including MRI, the specificity was less for dynamic TVUS only.

**Table 4:** Comparison of the Consecutive steps: History, Clinical Examination, Dynamic TVUS, and MRI in the Diagnosis of Endometriosis (n = 72)

Step	Sensitivity, %	Specificity, %	PPV, %	NPV, %	Accuracy, %	*P*
1. History	61.5	0	61.5	0	44.4	
2. History and clinical examination	58.6	0	70.8	0	47.2	NS
3. History, clinical examination, and dynamic TVUS	93.7	55.6	93.7	55.6	88.9	<.001
4. History, clinical examination, dynamic TVUS, and MRI	85.9	62.5	94.8	35.7	83.3	NS

Significance was calculated by comparison of step 2 to 1, step 3 to 2, and step 4 to 3. The added value of MRI compared to step 2 was significant (*P* < .001). NS indicates not significant (*P* > .05).

### *Comparison of the Consecutive Steps: History, Clinical Examination, Dynamic TVUS, and MRI in the Diagnosis of Deep Infiltrating Endometriosis*

In 59 of 72 patients, deep infiltrating endometriosis was confirmed by laparoscopy. In Table 5, the sensitivity, specificity, PPV, and NPV for each consecutive step in the diagnosis of deep infiltrating endometriosis are given. The sensitivity for the history, clinical examination, and dynamic TVUS was 93.2% (*P* < .001), whereas after inclusion of MRI findings, the sensitivity dropped to 88.1% (*P* = .375). As a consequence of the observational design of our study and the selection criteria for surgery, only patients with deep infiltrating endometriosis or a visual analog score of less than 7 underwent surgery; this approach explains why the NPV and specificity were either 0 or could not be calculated.

**Table 5:** Comparison of the Consecutive Steps: History, Clinical Examination, Dynamic TVUS, and MRI in the Diagnosis of Deep Infiltrating Endometriosis (n = 59)

Step	Sensitivity, %	Specificity, %	PPV, %	NPV, %	Accuracy, %	*P*
1. History	60.0	0	76.9	0	50.8	
2. History and clinical examination	59.3	0	86.5	0	54.2	NS
3. History, clinical examination, and dynamic TVUS	93.2	NN	100	0	93.2	<.001
4. History, clinical examination, dynamic TVUS, and MRI	88.1	NN	100	0	88.1	NS

Significance was calculated by comparison of step 2 to 1, step 3 to 2, and step 4 to 3. The added value of MRI compared to step 2 was significant (*P* < .001). NN indicates not a number; and NS, not significant (P > .05).

### *Prediction of the Correct Stage According to the Revised AFS Classification of Each Diagnostic Step Compared to Laparoscopic Findings*

Results of proper staging of endometriosis after each consecutive step in the diagnostic routine are given in Table 6. Correct staging after dynamic TVUS was 88.9% (64 of 72 patients); after MRI, it was 83.3%. Including dynamic TVUS findings only, underestimation by 1 stage performed better than after inclusion of MRI findings (5.6% versus 11.2%).

**Table 6:** Prediction of the Correct Stage of Endometriosis (Stage I–IV) According to the Revised AFS Classification of Each Diagnostic Step Compared to Laparoscopic Findings (n = 72)

Step	Correct Stage, % (n)	Overestimated by 1 Stage, % (n)	Underestimated by 1 Stage, % (n)	Overestimated by >1 Stage, % (n)	Underestimated by >1 Stage, % (n)
1. History	44.4 (32)	16.7 (12)	23.6 (17)	11.1 (8)	4.2 (3)
2. History and clinical examination	47.2 (34)	15.3 (11)	27.8 (20)	4.2 (3)	5.6 (4)
3. History, clinical examination, and dynamic TVUS	88.9 (64)	4.2 (3)	5.6 (4)	1.4 (1)	0 (0)
4. History, clinical examination, dynamic TVUS, and MRI	83.3 (60)	2.8 (2)	11.3 (8)	1.4 (1)	1.4 (1)

## Discussion

As endometriosis is a chronic and progressive disease, early diagnosis and proper staging are important for the patient and for the clinician to discuss and plan the required surgical procedures for treatment.^3^ Preoperative staging, using common diagnostic tools, such as the clinical history, physical examination, dynamic TVUS, and MRI, is very well feasible in deep infiltrating endometriosis. In this study, we evaluated the added value of each step in the diagnostic pathway for the assessment of (deep infiltrating) endometriosis in outpatient settings.

After the clinical history and pelvic examination, gynecologists were able to detect the correct stage of endometriosis according to the revised AFS classification in 47.2% of the patients. After the dynamic TVUS, this percentage increased to 88.9%. After MRI, this percentage decreased to 83.3%. This finding was due to the fact that MRI was not able to show (histologically confirmed) small rectal nodules in 7 of 72 patients: all between 1.5 and 2.5 cm.

The advantage of dynamic TVUS is the ability to evaluate the mobility of the pelvic organs and site‐specific pain.^15^, ^16^, ^17^, ^18^ This ability provides the gynecologist with additional information, which contributes to the assessment of the correct stage of disease. This factor may, in our opinion, explain why after the dynamic TVUS, the correct stage was more frequently predicted than after MRI. Although MRI gives a better overview of the abdomen, most deep infiltrating endometriosis lies in the pelvis.^21^

Different combinations of diagnostic tools have been evaluated recently. Marasinghe et al^22^ compared the history, pelvic examination, and mobility of ovaries to detect pelvic adhesions. They found sensitivity of 91% and specificity of 60.9% for identifying fixed ovaries secondary to endometriosis. Hudelist et al^8^ investigated the combination of clinical examination and TVUS for preoperative diagnosis of pelvic endometriosis and concluded that the combination of the physical examination accurately predicted the presence of endometriosis affecting the ovaries, vagina, rectum, uterosacral ligaments, rectovaginal septum, and pouch of Douglas in patients with suspected endometriosis. Abrao et al^9^ compared clinical examination, TVUS, and MRI for the diagnosis of deep infiltrating endometriosis and found that TVUS had better sensitivity and specificity in cases of deep retrocervical and rectosigmoid endometriosis compared to MRI and physical examination. However, Abrao et al^9^ focused on deep infiltrating endometriosis in the posterior compartment, and Hudelist et al^8^ compared TVUS to clinical examination only and without comparison to MRI, whereas Marasinghe et al^22^ only focused on pelvic adhesions.

To our knowledge, our study is the first prospective study to evaluate the added value of MRI after dynamic TVUS for staging endometriosis preoperatively and to test its usefulness in routine clinical practice. The most important factor for planning a laparoscopic resection of endometriosis is to identify all nodules caused by deep infiltrating endometriosis, since this step may influence the planning of the procedure.^3^

In an expert center, 88.9% of all patients with endometriosis can have the correct stage diagnosed at the first visit (Table 4), since TVUS is easily accessible in an outpatient setting. Also, TVUS and MRI are highly sensitive diagnostic tools for staging deep infiltrating endometriosis preoperatively. Although TVUS is less expensive and more accessible than MRI, our results were obtained by a single expert gynecologist for dynamic TVUS and a single radiologist for MRI. Ultrasound is much more operator dependent than MRI. Ultrasound is less sensitive for endometriosis beyond the field of view of the transvaginal transducer.^23^ It is also likely less sensitive for anterior compartment endometriosis.^23^ Routine MRI scans are therefore redundant and should only be performed when an extrapelvic location of deep infiltrating endometriosis is suspected or when a TVUS examination is not possible. This approach is in line with the findings of Turocy and Benacerraf.^24^

Several limitations of our study need to be considered. First, different scoring systems are proposed to document US findings regarding deep infiltrating endometriosis. Both the Enzian score^25^ and the scoring system used by Coccia and Rizello^26^ are more detailed than the revised AFS classification, as is the classification system developed by Exacoustos et al.^27^ However, these classification systems are still not widely used, so for the purpose of this study and comparison to the literature, classification by the revised AFS system was used, and deep infiltrating endometriosis nodules were noted separately.

Second, similar to the findings of Bazot et al,^7^ the prevalence of deep infiltrating endometriosis was particularly high, resulting in a particularly high rate of diagnosis by the clinical history and physical examination. This finding was inherent to the fact that the study was performed in a center with expertise in endometriosis. Another explanation for the high prevalence of deep infiltrating endometriosis in our study was that low‐grade endometriosis was treated conservatively.

We conclude that routine MRI after dynamic TVUS has no added value based on the following lines of evidence: First, the results in Table 4 clearly show that for diagnosis of pelvic endometriosis, inclusion of dynamic TVUS alone performed as well as after MRI. Second, the same conclusion can be drawn from Table 5 for diagnosis of deep infiltrating endometriosis. Third, dynamic TVUS performed even better at predicting the correct stage in patients predominantly affected by deep infiltrating endometriosis.

Our results clearly show that there is no substantial added value of routine MRI after dynamic TVUS for the preoperative staging of endometriosis. After the history and physical examination, dynamic TVUS and MRI both yield similar added value in preoperative staging of endometriosis with great overlap in clinical information. Both have their advantages and disadvantages, so choosing proper diagnostic imaging depends on the availability of an expert sonographer or MRI radiologist and on the anatomic site of interest based on the history and physical examination. Hopefully, this approach will eventually result in the reduction of costs for routine MRI scans, and more personalized preoperative counseling can be given to the patient.

## References

[1] Johnson NP , Hummelshoj L , Adamson GD , et al. Word Endometriosis Society consensus on the classification of endometriosis. Hum Reprod 2017; 2:315–324. 10.1093/humrep/dew293 27920089

[2] Eskenazi B , Warner ML . Epidemiology of endometriosis. Obstet Gynecol Clin North Am 1997; 24:235–258. 9163765 10.1016/s0889-8545(05)70302-8

[3] Meuleman C , Vandenabeele B , Fieuws S , Spiessens C , Timmerman D , D'Hooghe T . High prevalence of endometriosis in infertile women with normal ovulation an normospermic partners. Fertil Steril 2009; 92:68–74. 18684448 10.1016/j.fertnstert.2008.04.056

[4] Hadfield R , Mardon H , Barlow D , Kenndy S . Delay in the diagnosis of endometriosis: a survey of women from the USA and UK. Hum Reprod 1996; 114:878–880. 10.1093/oxfordjournals.humrep.a019270 8671344

[5] Husby GK , Haugen RS , Moen MH . Diagnostic delay in women with pain and endometriosis. Acta Obstet Gynecol Scand 2003; 827:649–653. 10.1034/j.1600-0412.2003.00168.x 12790847

[6] Ballard K , Lowton K , Wright J . What's the delay? A qualitative study of women's experiences of reaching a diagnosis of endometriosis. Fertil Steril 2006; 86:1296–1301. 17070183 10.1016/j.fertnstert.2006.04.054

[7] Bazot M , Lafont C , Rouzier R , Roseau G , Thomassin‐Naggara I , Daraï E . Diagnostic accuracy of physical examination, transvaginal sonography, rectal endoscopic sonography, and magnetic resonance imaging to diagnose deep infiltrating endometriosis. Fertil Steril 2009; 92:1825–1833. 19019357 10.1016/j.fertnstert.2008.09.005

[8] Hudelist G , Oerwinkler KH , Singer CF , et al. Combination of transvaginal sonography and clinical examination for preoperative diagnosis of pelvic endometriosis. Hum Reprod 2009; 24:1018–1024. 19202143 10.1093/humrep/dep013

[9] Abrao MS , Goncalves MO , Dias JA Jr , Podgaec S , Chamie LP , Blasbalg R . Comparison between clinical examination, transvaginal sonography and magnetic resonance imaging for the diagnosis of deep endometriosis. Hum Reprod 2007; 22:3092–3097. 17947378 10.1093/humrep/dem187

[10] van der Wat J , Kaplan MD . Modified virtual colonoscopy: a noninvasive technique for the diagnosis of rectovaginal septum and deep infiltrating pelvic endometriosis. J Minim Invasive Gynecol 2007; 14:638–643. 17848328 10.1016/j.jmig.2007.03.010

[11] Bazot M , Darai E , Hourani R , et al. Deep pelvic endometriosis: MR imaging for diagnosis and prediction of extension of disease. Radiology 2004; 232:379–389. 15205479 10.1148/radiol.2322030762

[12] Hudelist G , English J , Thomas AE , Tinelli A , Singer CF , Keckstein J . Diagnostic accuracy of transvaginal ultrasound for non‐invasive diagnosis of bowel endometriosis: a systematic review and meta‐analysis. Ultrasound Obstet Gynecol 2011; 37:257–263. 20954166 10.1002/uog.8858

[13] Hudelist G , Fritzer N , Staettner S , et al. Uterine sliding sign: a simple sonographic predictor for presence of deep infiltrating endometriosis of the rectum. Ultrasound Obstet Gynecol 2013; 41:692–695. 23400893 10.1002/uog.12431

[14] Reid S , Lu C , Casikar I , et al. The prediction of pouch of Douglas obliteration using offline analysis of the transvaginal ultrasound “sliding sign” technique: inter‐ and intra‐observer reproducibility. Hum Reprod 2013; 28:1237–1246. 23482338 10.1093/humrep/det044

[15] Guerriero S , Ajossa S , Gerada M , Virgilio B , Angioni S , Melis GB . Diagnostic value of transvaginal “tenderness‐guided” ultrasonography for the prediction of location of deep endometriosis. Hum Reprod 2008; 23:2452–2457. 18664469 10.1093/humrep/den293

[16] Guerriero S , Condous G , van den Bosch T , et al. Systematic approach to sonographic evaluation of the pelvis in women with suspected endometriosis, including terms, definitions and measurements: a consensus opinion from the International Deep Endometriosis Analysis (IDEA) group. Ultrasound Obstet Gynecol 2016; 48:318–323. 27349699 10.1002/uog.15955

[17] Okaro E , Condous G , Khalid A , et al. The use of ultrasound‐based “soft markers” for the prediction of pelvic pathology in women with chronic pelvic pain: can we reduce the need for laparoscopy? BJOG 2006; 113:251–256. 16487194 10.1111/j.1471-0528.2006.00849.x

[18] Dunselman GA , Vermeulen N , Becker C , et al. ESHRE guideline: management of women with endometriosis. Hum Reprod 2014; 29:1353–1359. 24435778 10.1093/humrep/det457

[19] American Society for Reproductive Medicine . Revised American Society for Reproductive Medicine classification of endometriosis: 1996. Fertil Steril 1997; 67:817–821. 9130884 10.1016/s0015-0282(97)81391-x

[20] Fernando S , Qian Soh P , Cooper M , et al. Reliability of visual diagnosis of endometriosis. J Minim Invasive Gynecol 2013; 20:783–789. 24183270 10.1016/j.jmig.2013.04.017

[21] Chapron C , Fauconnier A , Vieira M , et al. Anatomical distribution of deeply infiltrating endometriosis: surgical implications and proposition for a classification. Hum Reprod 2003; 18:157–161. 12525459 10.1093/humrep/deg009

[22] Marasinghe JP , Senanayake H , Saravanabhava N , Arambepola C , Condous G , Greenwood P . History, pelvic examination findings and mobility of ovaries as a sonographic marker to detect pelvic adhesions with fixed ovaries. J Obstet Gynaecol Res 2014; 40:785–790. 24738122 10.1111/jog.12234

[23] Nisenblat V , Prentice L , Bossuyt PMM , Farquhar C , Hull ML , Johnson N . Combination of the non‐invasive tests for the diagnosis of endometriosis. Cochrane Database Syst Rev 2016; 7:CD012281. 27405583 10.1002/14651858.CD012281 PMC6953325

[24] Turocy JM , Benacerraf BR . Transvaginal sonography in the diagnosis of deep infiltrating endometriosis: a review. J Clin Ultrasound 2017; 45:313–318. 28414865 10.1002/jcu.22483

[25] Di Paola V , Manfredi R , Castelli F , Negrelli R , Mehrabi S , Pozzi Mucelli R . Detection and localization of deep endometriosis by means of MRI and correlation with the ENZIAN score. Eur J Radiol 2015; 84:568–574. 25604906 10.1016/j.ejrad.2014.12.017

[26] Coccia ME , Rizello F . Ultrasonographic staging: a new staging system for deep endometriosis. Ann NY Acad Sci 2011; 1221:61–69. 21401631 10.1111/j.1749-6632.2011.05951.x

[27] Exacoustos C , Malzon M , Di Giovanni A , et al. Ultrasound mapping system for the surgical management of deep infiltrating endometriosis. Fertil Steril 2014; 102:143–150. 24794315 10.1016/j.fertnstert.2014.03.043

